# Where’s the leadership? Future commitments of Unicef and WHO for global child health

**DOI:** 10.1136/bmj.k3219

**Published:** 2018-07-30

**Authors:** Anthony Costello, Stefan Peterson, Kumanan Rasanathan, Bernadette Daelmans, Rajiv Bahl

**Affiliations:** 1 Department of Maternal, Newborn, Child and Adolescent Health, World Health Organization, Geneva, Switzerland; 2 Health Section, Unicef, New York, USA; Correspondence to: A Costello cihdcostello@gmail.com

## Abstract

**Anthony Costello and colleagues** explain how WHO and Unicef are working closely together to support countries to achieve universal health coverage for children

Country leadership and national tailored strategies for child survival, health, and development are essential to achieve universal health coverage for children. As the principal UN agencies responsible for child health, Unicef and the World Health Organization have a duty to tackle the challenges in global child health leadership identified by the strategic review *Towards a Grand Convergence for Child Survival and Health* 1 and a recent report by USAID,^2^ *Mapping Global Leadership in Child Health*. These reports highlight inadequate coordination of global leadership that has led to fragmentation, weakened accountability, and reduced the effect on children’s lives.

During implementation of the Integrated Management of Childhood Illness strategy (IMCI) health systems challenges, including drug supply, staff recruitment, and supervision, proved more difficult than envisaged. IMCI programme plans focused largely on staff training and skills to the detriment of the community component. IMCI may have been accepted as a strategy but insufficient attention was given to turning the strategy into an integrated programme.

Based on these issues and the findings of these reports, we summarise the main challenges and areas for action by WHO and Unicef.

## Challenges

We have identified five main challenges to reaching global child health goals outlined in the UN sustainable development goals (SDGs) and the Global Strategy for Women’s, Children’s and Adolescents’ Health (2016-2030) (table 1).

**Table 1:** Recommendations of the strategic review of IMCI^1^

Problem	Recommended solution
Fragmentation of global child health strategies undermines programming and limits effect	WHO-Unicef issue joint statement to reposition IMCI, child health
	Partners consolidate around one leadership body
	Country stakeholders advocate for high level representation in coordination mechanisms
Inadequate funding and delivery to marginalised populations	Global partners develop innovative strategies to target poor populations and support removal of user fees
	Country leaders mobilise support and resources and use Global Financing Facility investment cases to develop ambitious, costed plans
	WHO-Unicef develop less resource intensive training
Evidence is not systematically generated, captured, and integrated into policy and programming.	WHO-Unicef establish a global expert advisory group to gain consensus on state of the art recommendations
	Partners create an online resource hub and forum
	Regional actors provide technical and policy support Countries use implementation science and facilitate shared learning among district teams
Strategies are insufficiently tailored to country context and tools need to be easier to use	WHO-Unicef harmonise tools into a one flexible, adaptable set with input from users and design specialists
	Expert advisory group recommends strategies to build on country strengths (private sector, community, etc)
	Combined approach for facility, systems, community
Lack of accountability and corresponding need for clear targets and strong monitoring	WHO-Unicef establish a joint leadership process to develop and adopt clear IMCI targets, alongside global accountability processes
	Partners strengthen country capabilities to routinely monitor and evaluate progress using scorecards
	Countries scale up monitoring alongside improved community engagement, using data to enhance accountability

Additional details and process indicators associated with each recommendation are provided in the annex to the strategic review^1^

The vision of the SDGs for women and children requires adequate funding and provision for service delivery to poor and marginalised populations. This must be supported by clear, long term commitments from governments and international donors to reach the necessary investment to achieve universal health coverage. Supply-side measures alone are insufficient; investment is also required in community empowerment and rights based approaches.

The implementation tools must also be versatile. They must be easily adapted and meet an array of programme needs in different countries for quality as well as coverage of interventions and services. A joint initiative between WHO and Unicef, “Child Health Redesign” has started to collate the ideas of many stakeholders to this end. A review paper and expert meeting is scheduled for autumn 2018.

The ICMI review concluded that the process of evidence gathering, synthesis, and assessment has so far not been sufficiently systematic, leading to perceived gaps in integration of new evidence into policy and programming. The broader maternal and child health agenda can benefit from the example of immunisation, where global organisations and donors are guided by recommendations of a scientific and technical advisory group of experts (STAGE) in formulating policy.

Fragmentation of global child health programmes may have also limited their effect on the lives of children and their families. Global agencies have spawned a dizzying array of global initiatives in the past decade with the good intention of increasing the coverage of life saving interventions. However, too many initiatives hinder coherence in technical and funding support and clarity for country leaders and ministries of health.^3^

The final challenge is a lack of accountability, internationally and nationally. Clear targets, appropriate monitoring mechanisms with timely availability of usable data, and institutional structures for remedial action at national, regional, and global levels are lacking.

## Potential solutions and barriers to success

Universal health coverage is one of the three key components of the new WHO strategy under the director general, Tedros Adhanom Ghebreyesus. Investment in and management of health systems are central to success. A review meeting in Almaty will examine these issues in October 2018, the 40th anniversary of the Alma-Ata conference on primary healthcare. The WHO-Unicef child health redesign initiative will draw on these discussions.

Programmatic and systems thinking will underlie all of WHO’s and Unicef’s work towards universal health coverage. We will support countries to develop results frameworks that look at process, management, and outcomes. Indicators of progress will be monitored closely in a way that was not possible to achieve with IMCI. Furthermore, the community component must be strengthened in our redesign of programmes.

We are under no illusions about the challenge in achieving the sustainable development goals for children. Three important barriers are a lack of investment and finance in country programmes, conflict and fragile states, and weak national leadership and commitment to maternal and child health programmes. In many countries both political will and investment in reproductive, maternal, and child and adolescent health programmes are inadequate.

## Primacy of country leadership

Country leadership and management are critical. The countries with the greatest reductions in child mortality over the past quarter century are those whose leaders and communities are committed to policies to improve social, economic, and environmental determinants of health, while at the same time tailoring health promotion, prevention, and treatment programmes to overcome specific bottlenecks within health systems and across sectors.^4^ ^5^ The role of global agencies is to support local people and their leadership to define and implement solutions.

In February 2017, WHO, Unicef, UNFPA, and partners from all stakeholder groups launched the Network for Improving Quality of Care for Maternal, Newborn and Child Health. Supported by a learning system to promote implementation principles, quality standards, and evidence based interventions, governments and partners from the countries****in the network collaborate to build quality in health services and strengthen the global evidence of what works, where, when, and how.^6^ It aims to institutionalise quality improvement and control within national health systems and act as a pathfinder for more people centred care and to achieve universal health coverage.

The network brings together a wide range of stakeholders from government, civic society organisations, national academia, healthcare, and the private sector. WHO and Unicef intend to extend this programme and share important lessons from it.

For presidents and prime ministers, we should echo the wise words of Awa Coll-Seck, former Senegalese minister of health and now minister of state, at a recent child health review meeting in Senegal: “Human capital is now at the very core of our long-term plan … we had to fight with those who saw the long-term plan as purely economic.”

Although the strategic review showed that both IMCI and integrated community case management of childhood illness (iCCM) were highly appreciated by healthcare providers, the same respondents thought end user guidelines and tools were designed too narrowly to fit diverse country contexts, too cumbersome to adapt, and too costly to fully implement. Furthermore, country staff sometimes resented global organisations’ prescriptive attitudes towards implementation: as one policy maker said, “[Partners] think they know much better than the country—which is unlikely.”

Below we lay out our commitments to tackle these challenges and enact the recommendations to enable the survival, health, and development of all children worldwide.

## Unicef-WHO leadership strategy: five key areas

We identified five key areas for strengthening Unicef and WHO’s collaboration, with specific commitments to realise child health targets set out in the sustainable development goals. A holistic view of child health will be reasserted to tackle the determinants of child health through multisectoral action, including early childhood development, nutrition, injury prevention, environmental threats, and adolescent health and engagement.

### Increase coverage of child health interventions and reduce inequities

Up to half of improvements in child survival are a result of actions beyond the health sector, and enabling children to thrive is impossible without action on health determinants led by other sectors.^5^ Reaching the unreached populations requires a mixed portfolio of multisectoral investment to ensure policies and services enable families to access resources. We propose to remedy the gap in our technical help to countries and support multisectoral efforts under the Every Woman Every Child (EWEC) partnership, including in governance, financing, joint accountability, and prospective evaluation of impact. We will work across sectors on promoting breastfeeding, prevention of stunting, early childhood development, and adolescent health, including areas such as non-communicable diseases and mental health. We will seek stronger partnerships between governments and non-state actors and implement actions recommended by the high level working group on the health and human rights of women, children and adolescents.^7^

WHO and Unicef are only part of the United Nations picture. The H6 group (WHO, Unicef, UN Women, UNAIDs, UNFPA, and UN Foundation) together with the Global Fund, Global Alliance Vaccines Initiative (GAVI), Global Finance Facility (GFF), and the World Bank all have important and complementary roles. We shall work closely with the Global Finance Facility (GFF) at the World Bank, established to support reproductive, maternal, and child health programmes. The GFF was set up to address an annual shortfall of $33bn a year for these programmes. It aims to support over 50 countries and has targeted a $2bn investment in the first wave countries.

But we have a long journey to ensure that the most marginalised families and communities have the rights, voice, and justice required to achieve the sustainable development goals. The UN secretary-general has made women and community empowerment a key focus for the EWEC transform agenda. Meta-analysis of trials leading to a formal guideline from WHO has shown that community mobilisation through women’s groups and participatory learning and action cycles can substantially improve newborn survival in marginalised and rural communities.^8^

WHO has also reviewed evidence to identify other effective interventions to strengthen individual, family, and community capacity to improve home care practices and care seeking for women and newborn children. The voices of women and communities are key inputs to long term solutions. WHO has published a new toolkit on how to conduct participatory planning at the district level, a process whereby civil society, health committees, political leaders, and community groups can provide input and feedback to district health plans.

We shall collaborate to strengthen the interactions between health services, local authorities, and communities to overcome barriers to universal health coverage. This will not be easy, and critics might suggest we have failed in the past. To address this WHO and Unicef commit to work with innovation partners on technology to improve social and citizen accountability for their health services. The UN secretary general has also launched a reform programme to coordinate UN inputs in countries with a strengthened system of resident coordinators, who will ensure that different UN agencies in the same country collaborate and work to a common plan agreed with national governments. In addition, the cross-agency commitments of the sustainable development goals make us jointly accountable.

### Ensure versatile implementation tools for easy adaptation to all country contexts

Global agencies must be thoughtful and restrained in supporting national leaders and ministries of health. Health systems are built from the ground up, with strategies made to fit to epidemiological and economic contexts. Global partner support to countries must be similarly diversified. For example, children in countries with large private sectors might benefit from their governments adopting evidence based strategies for engaging with drug shops, dispensaries, and social franchises. Countries with large cadres of community health workers can build on this capacity for scaling up promotive, preventive, and curative activities at community level. Although global public goods such as strategies, guidelines, and tools are useful, countries need support in adapting these for their requirements.

Over the next two years WHO and Unicef will work together to redesign the global approach to child health, with implications for content and presentation of all our child health strategies and tools. The redesign is about how to link the three levels of health worker skills, health systems, and community engagement. A key element of overall WHO strategy is to increase the number of people with “healthy lives,” and strengthening the community component of reproductive, maternal, and child health will be central to its success.

The interpretation of “child’ will not be limited to the first 5 years. New ways of packaging global guidelines will better serve country level implementers based on principles of flexibility, adaptability, and end user design. Our guideline dissemination strategy will encompass multiple platforms, both digital and published, to address different audiences and needs and ensure that information to help children survive and thrive is easily accessed.^9^

We will produce an updated, refined, and harmonised newborn and child health guidance package, including guidelines on promotion, prevention, assessment, and treatment. This package will include adaptable tools across all levels of care to ensure a high standard of care across diverse health systems and to respond to the changing epidemiology and aetiology of major childhood killers, as well as their distribution.

In particular, given the increasing concentration of child mortality and morbidity in humanitarian and fragile settings, we shall provide greater guidance on the delivery of services in these contexts. Currently, WHO and Unicef support healthcare in seven countries with grade 3 emergencies (Bangladesh Rohingya, Democratic Republic of Congo, Iraq, Nigeria, Somalia, South Sudan, Syria, and Yemen), 10 countries with grade 2 emergencies, and 11 with grade 1. Sixty one countries have a score of 80 or above on the Fragile States Index.^10^ We shall work with partners such as Médecins Sans Frontières and UNHCR to provide the best evidence and guidelines for work in challenging and humanitarian settings. Implementation models require more evidence. We must avoid situations such as mobile hospitals set up for Ebola outbreaks having no antibiotics to treat common childhood infections, malnutrition, or malaria. We cannot provide integrated support if humanitarian and reproductive, maternal, and child health teams are siloed. And we must bring pressure to bear, through the UN Security Council, on combatants that increasingly target health workers and health facilities.

### Adopt a systematic process to generate and capture evidence for policy and programming

To provide a regular, rigorous, realistic review of the evidence for new interventions and programmes at scale, we shall launch a global expert advisory group in 2018 to convene annually at WHO to advise on child health policies and strategies. Efforts to generate country specific evidence and introduce relevant innovations are already under way. WHO and Unicef are developing a scientific and technical advisory group of experts (STAGE) for maternal, child, and adolescent health programmes, with independent experts providing advice and scrutiny to the organisations’ director generals and to governments.

### Provide integrated technical and financial support to country programmes and reduce fragmentation

A lack of unified global leadership has resulted in fragmented strategies, large scale inefficiencies, and missed opportunities for synergy, reducing the effect on child health outcomes.^2^ The schism between IMCI and iCCM, the separation of maternal and newborn health initiatives, the failure to implement interventions to promote family and community practices, and the inability to coordinate policies and interventions beyond the health sector are a few examples. As representatives of the principal UN agencies responsible for the health of the child, we will seek to tackle this problem as part of the secretary general’s call to integrate country support through “One United Nations.”

The sustainable development goals call for much greater collaboration between all UN health related agencies, as well as the many global and national organisations in health. Within the UN family, we meet our partners at UNAIDS, UNFPA, UN Women, and the World Bank under the H6 umbrella. Together, we aim to harmonise support in the form of joint proposals and field based action, in concert with country investment under national plans. Real cross-agency collaboration will reinforce the leadership of ministries of health and enable all to work together in support of one country plan. The concept of a single investment plan for reproductive, maternal, child, and adolescent health to which all funders subscribe has proved difficult in practice but is the only viable model.

Recent action on newborn health shows what is possible. The Every Newborn Action Plan, coordinated by Unicef and WHO catalysed commitment and action for maternal and newborn health at global, regional, and country levels. H6 partner coordination was strengthened, including in Africa, the Middle East, and Asia. By January 2017, 48 countries and territories had strengthened maternal and newborn plans and 40 countries had set targets for newborn mortality rates. These included 19 of the 20 countries with the highest burden of newborn mortality. In addition, 16 countries developed subnational plans and 21 countries had completed costing of their national plans.

### Collaborate on indicators and frameworks to ensure accountability

Accountability is about how countries can monitor, review, and act on what is happening in child health, whether the outcome is preventable newborn deaths, child obesity, or adolescent road traffic injuries. Country capacity for monitoring and evaluation is critical, alongside strengthening of national structures and processes for review and action.

Since the Commission on Information and Accountability for Women’s and Children’s Health made its recommendations in 2011, efforts to strengthen accountability for results and resources for maternal, newborn, and child health have been made at all levels. At present, the emphasis on universal health coverage advanced by the WHO director general offers an opportunity to advance practical strategies to reach women, children, and poor populations and to galvanise political leaders to hold stakeholders to account.

The challenge is huge. Data systems are weak, quality of data variable, and analyses open to criticism. One critical area of collaboration is to effectively monitor global strategy targets and core indicators. The WHO Global Health Observatory brings together country data in an open and accessible database for countries. WHO and Unicef will work with global monitoring bodies such as the observatory, Countdown to 2030, and the Institute of Health Metrics and Evaluation, as well as research funders, to strengthen data collection and analysis. We shall also provide a global policy database and work to improve the definition and collection of core indicators with international researchers and national governments. We have already set up a group of independent experts to review approaches to Mother and Newborn Information to Track Outcomes and Results (MONITOR). This group will harmonise maternal and newborn measurement efforts and provide guidance for improving data collection and national capacities, based on the latest evidence. We plan to establish a similar group for child and adolescent data. The UN EWEC strategy, which was coordinated by WHO and Unicef, has 15 core indicators being monitored through annual reports and reviewed by an Independent Advisory Panel. All data are available through a web portal (https://www.everywomaneverychild.org/2017/07/17/progress-report-on-the-ewec-global-strategy-for-womens-childrens-and-adolescents-health/).

## IMCI: what now?

The IMCI strategy and its younger sibling, iCCM, have enjoyed widespread popularity thanks to their holistic view of the child and comprehensive inclusion of interventions at facility, health systems, and community level. Over 100 countries have adopted IMCI, and at least 45 countries in malaria endemic regions have adopted iCCM.

In our strategic review we heard from policy makers, child health programme managers, and service providers around the world, who praised IMCI for its simplicity and comprehensiveness. As one Nigerian nurse put it: “IMCI is a strategy that really can help children.” However, we now need to reposition IMCI and child health more broadly with respect to the goals in the Global Strategy for Women’s, Children’s and Adolescents’ Health (2016-2030), and to reflect the fact that declines in deaths from infectious diseases are accompanied by relative or absolute increases in obesity, asthma, injury, and maltreatment. Most children now live in urban settings so our strategy must also reflect this shift.

Although it is hard to measure cause and effect, a recent Cochrane review suggests IMCI contributed to reductions in child mortality in countries where it was implemented^11^; iCCM has supplemented these gains by expanding access to underserved populations. However problems identified by the multicountry evaluation of IMCI 10 years ago—“difficulties in expanding the strategy at national level while maintaining adequate intervention quality,” and failure to account for the “full weight of health systems inadequacies,”—remain today.^12^

Furthermore, commitment and funding for IMCI has waned over the past two decades, with attention having shifted from integrated care to specific interventions such as immunisation and treatment of a few communicable diseases. Whether we repackage IMCI or rebrand a new global child health strategy remains an open question. Although the “brand” has strong, positive recognition in countries around the world, its name refers to “case management” and not the wider vision of child health.

The need for political commitment, investment, and coordinated programmes to improve child health is not in doubt. A redefined strategy will take “ownership” of this broader vision of child health, covering both the sick and well child, from birth to adolescence, and including promotion, prevention, and treatment activities. It will be much better integrated with maternal health, as evidence shows that half of newborn survival derives from interventions delivered to mothers during pregnancy or delivery.^13^

WHO and Unicef can—and must—do better. We have laid out key actions for the future and a process of accountability to monitor and review whether we achieve our aims (box 1) to convene, collaborate, and catalyse country action for the world’s children.

Box 1 **Holding Unicef and WHO to account**

Our commitments to these five key areas are measurable. We should be held to account. We propose that our progress is independently reviewed every two years starting in 2019. Indicators of progress might include some, or all, of the following:

**Coverage**

- Equity measures are incorporated into routine reporting
- Integrated guidance on participatory planning in districts is widely used in high burden countries and is raising coverage in marginalised populations

**Redesign**

•Joint new child health redesign package and process is set up that included guidance on multisectoral activities
•Flexible new child health guidelines (0-18 years) are available on a new user friendly platform

**Quality**

- The Network for Improving Quality of Care for Maternal, Newborn and Child Health is expanded and evaluated
- Country level workshops support leaders to design country specific child health packages and use redesign guidelines
- Formal and regular learning and review systems are established to integrate latest scientific evidence into child health guidelines

**Integrated technical support**

- WHO-Unicef workplans in high burden countries are integrated and synergistic
- These plans coordinate with the overall WHO biennium and Unicef plans

**Joint framework for accountability**

•WHO and Unicef work with global monitoring bodies such as the WHO Global Health Observatory, Countdown to 2030, the Institute of Health Metrics and Evaluation to integrate datasets available in real time to countries
WHO and Unicef have strengthened regional and country data collection and analysis for women’s, children’s, and adolescent health

**Key messages**

- Many millions of children still do not survive, thrive, or live in healthy environments
- Global agencies must strengthen their effectiveness at country level to provide consistent, coherent, and integrated advice to ministries of health
- WHO and Unicef have brought their child health teams much closer together with new global initiatives on redesigning child health, quality of care for mothers and children, and for community based mobilisation and care
- Future technical guidance will be unified, with each agency taking responsibility for areas in which it has a comparative advantage

## Notes

Contributors and sources: This article is based on findings from a global review of Integrated Management of Childhood Illness (IMCI) and integrated Community Case Management (iCCM) commissioned by WHO. AC conceived of this paper and wrote the first draft and subsequent drafts were completed in iteration with the other authors. All authors contributed intellectual content, edited the manuscript, and approved the final version for submission. The authors alone are responsible for the views expressed in this article, which does not necessarily represent the views, decisions, or policies of the institutions with which the authors are affiliated.

Provenance and peer review: Commissioned; externally peer reviewed.

This article is part of a series based on findings from a global review of Integrated Management of Childhood Illness funded by the Bill and Melinda Gates Foundation. Open access fees were funded by the Health Systems Research Unit, South African Medical Research Council. *The BMJ* peer reviewed, edited, and made the decision to publish the article with no involvement from the Medical Research Council.
